# The Effect of High Intensity Interval Exercise on Postprandial Triacylglycerol and Leukocyte Activation – Monitored for 48h Post Exercise

**DOI:** 10.1371/journal.pone.0082669

**Published:** 2013-12-09

**Authors:** Brendan Morris Gabriel, Jamie Pugh, Valerie Pruneta-Deloche, Philippe Moulin, Aivaras Ratkevicius, Stuart Robert Gray

**Affiliations:** 1 Institute of Medical Sciences, University of Aberdeen, Aberdeen, United Kingdom; 2 Endocrinology Department, Hopital Louis Pradel, University Lyon, Lyon, France; 3 School of Sport, Exercise and Health Sciences, Loughborough University, Loughborough, United Kingdom; University of Bath, United Kingdom

## Abstract

Postprandial phenomenon are thought to contribute to atherogenesis alongside activation of the immune system. A single bout of high intensity interval exercise attenuates postprandial triacylglycerol (TG), although the longevity and mechanisms underlying this observation are unknown. The aims of this study were to determine whether this attenuation in postprandial TG remained 2 days after high intensity interval exercise, to monitor markers of leukocyte activation and investigate the underlying mechanisms. Eight young men each completed two three day trials. On day 1: subjects rested (Control) or performed 5 x 30 s maximal sprints (high intensity interval exercise). On day 2 and 3 subjects consumed high fat meals for breakfast and 3 h later for lunch. Blood samples were taken at various times and analysed for TG, glucose and TG-rich lipoprotein (TRL)-bound LPL-dependent TRL-TG hydrolysis (LTTH). Flow cytometry was used to evaluate granulocyte, monocyte and lymphocyte CD11b and CD36 expression. On day 2 after high intensity interval exercise TG area under the curve was lower (P<0.05) (7.46±1.53 mmol/l/7h) compared to the control trial (9.47±3.04 mmol/l/7h) with no differences during day 3 of the trial. LTTH activity was higher (P<0.05) after high intensity interval exercise, at 2 hours of day 2, compared to control. Granulocyte, monocyte and lymphocyte CD11b expression increased with time over day 2 and 3 of the study (P<0.0001). Lymphocyte and monocyte CD36 expression decreased with time over day 2 and 3 (P<0.05). There were no differences between trials in CD11b and CD36 expression on any leukocytes. A single session of high intensity interval exercise attenuated postprandial TG on day 2 of the study, with this effect abolished by day 3.The reduction in postprandial TG was associated with an increase in LTTH. High intensity interval exercise had no effect on postprandial responses of CD11b or CD36.

## Introduction

Cardiovascular disease (CVD) is the leading cause of death worldwide, and is becoming more prevalent [1]. The most common CVD is coronary artery disease (CAD), which has atherosclerosis at the centre of its pathology. In recent years it has been established that atherosclerosis is a chronic inflammatory condition with immune cells present in the artery wall and plaque itself (for review see 2). Whilst there is clear data linking fasting LDL levels to atherogenesis [3] fasting triacylglcerol (TG) levels have been found to be a poor, with non-fasting TG a strong, independent predictor of atherosclerosis and CAD [4]. Furthermore, as humans spend the majority of their day in a postprandial state it has been suggested that this postprandial phase is a strong contributor in atherogenesis [5].

The precise mechanism linking postprandial TG levels with atherosclerosis are unknown. It is thought to result from a series of linked events including the generation of free radicals and activation of the immune system leading to endothelial dysfunction, an early event in the development of atherosclerosis [6]. Even a single high fat meal induces endothelial dysfunction, the magnitude of which associates strongly with the magnitude of postprandial TG [7]. Support for the role of oxidative stress as a cause of endothelial dysfunction has come from studies demonstrating that the consumption of antioxidants (Vitamin C) can attenuate the magnitude of postprandial endothelial dysfunction [8-10]. Additionally, following a high fat meal, the changes in circulating TG positively correlate with leukocyte O_2_- production and endothelial dysfunction [11], indicating concurrent activation of the immune system. 

Indeed several studies have shown that TG rich lipoproteins (TRL) activate monocytes (increased CD11b) and to a lesser extent neutrophils (increased CD11b and CD66b) via uptake of TG [12]. These findings have recently been extended by Gower et al. [13] who found that after a high fat meal monocytes internalise lipid, upregulate CD11c and increase adhesion to VCAM-1. This leukocyte activation has several consequences including an increase in pro-inflammatory cytokine production, oxidative stress, adhesion, activation of endothelial cells and ultimately an increase in migration of leukocytes and lipoproteins to the sub endothelium (for review see 14). Leukocyte activation can also lead to the uptake of oxidised plasma low density lipoprotein (oxLDL) by monocyte derived macrophages in the vascular wall, a well established step in the development of atherosclerosis [15]. The scavenger receptor CD36 has been shown to be central in facilitating monocyte/macrophage uptake of oxLDL [16,17], and appears to be linked to atherosclerosis [18][19]. The effect of a high fat meal on leukocyte CD36 expression, however, remains to be established. 

Several investigators have reported the beneficial effects of a single bout of exercise on postprandial TG with both endurance exercise and high intensity interval (HIIE) being demonstrated to reduce postprandial TG the following day [20] [21] [22] [23], with the latter attracting interest due to its relative time efficiency [24]. What remains to be established is whether these effects remain on the second day after HIIE, information which will have clear implications for the required frequency of such exercise and exercise prescription recommendations. Furthermore, the mechanisms underlying the reductions in TG after HIIE have yet to be investigated. Potential candidates are: either a reduction in hepatic VLDL secretion or an increase in lipoprotein lipase (LPL) activity. In our previous work we have shown that HIIE does not affect plasma β-hydroxybutyrate, a marker of hepatic fatty acid oxidation changes in which would shift hepatic fatty acid portioning and alter VLDL synthesis, making an increase in LPL activity the likely candidate [23]. 

The aim of this study was to determine whether the attenuation in postprandial TG remained 2 days after a bout HIIE. A secondary aim of the study was to determine the effects of HIIE on leukocyte activation. A further aim was to determine the mechanisms underlying the reduction in postprandial lipaemia observed after HIIE by measuring TRL-bound LPL dependent TRL-TG hydrolysis (LTTH). 

## Materials and Methods

### Participants

Eight healthy male participants took part in this study (age; 25±4 years, body fat %; 11.7±4.1%, weight; 72.9±15.3 kg, height; 1.77±0.13 m). All participants were regularly physically active but none were specifically trained. Exclusion criteria for volunteers included a history of cardiovascular disease, smoking, hypertension (systolic/diastolic blood pressure >140/90 mmHg), diabetes, obesity (BMI >30 kg/m^2^) or subjects with any form of musculoskeletal injury. The power of the current proposal has been based on postprandial TG. The analysis is based on a repeated measures design with 2 treatment groups (control and high intensity). Using the reduction in TG AUC 10 participants would provide 80% statistical power at α=0.05 to detect a difference in means of 3.26 mmol/l/7h (observed in our previous work) assuming a common standard deviation of 3.5. Due to drop-out of 2 participants only eight are included in the final analysis. 

### Ethics statement

The study conformed to current local guidelines, the declaration of Helsinki and was approved by the University of Aberdeen College Ethical Review Board. All participants were fully informed of the aims, risks and discomfort associated with the investigation before providing written informed consent.

### Anthropometric measurements

Height was measured to the nearest 0.5 cm using a stadiometer (Holtain Ltd., Crymych, Dyfed, Wales, UK). Weight was measured to the nearest 0.1 kg using a weighing scale (Ohaus champ 2, Ohaus UK Ltd., Leicester, England). Skinfold thickness was measured with callipers (Idass, Harpenden skinfold callipers, England, UK.) at 4 sites (Bicep, triceps, sub scapula & supra iliac) to the nearest 0.1 mm on the right side of the body. Percentage body fat was calculated using standard methods [25]. 

### Experimental Protocol

Participants completed two three day trials in a randomized order. On day 1 (14:00 hours) subjects rested for 30 min (Control) or performed 5 x 30 sec maximal sprints with 4 min recovery between each sprint (HIIE). On day 2 (beginning at 9:00 hours) subjects arrived after an overnight fast and consumed a high fat meal for breakfast and 3 h later for lunch, this was repeated for day 3. Each trial was separated by at least 7 days. Participants were instructed not to ingest alcohol or caffeine in the 24 h period prior to day 1 up until the end of day 3. During this time they were also asked to refrain from exercise or strenuous physical activity other than that of the trials. Subjects were also asked to record their diet 24 hours before day 1 and during the trial and replicate this during the subsequent visit.

### Day 1

#### High Intensity Interval Exercise

During the HIIE trials exercise was performed on a cycle ergometer (Monark 894, Wingate bike, UK). Subjects performed a 4 min warm-up with no load and then performed a 30 s maximal sprint against a load of 7.5% body weight followed again by unloaded cycling for 4 min. During the warm-up and unloaded cycling phases participants were asked to maintain a cadence of ~70 rpm and accelerate after the 7.5% body weight resistance was applied. This sprint exercise was repeated a further four times, with 4 min unloaded cycling in between each sprint. During each 30 s sprint average power (Watts), peak power (Watts) and peak pedal rate (RPM) were recorded. Energy expenditure was estimated during the high intensity trial using the mean power output during the 30 s sprint and an estimate of mechanical efficiency of 8.5% [16]. 

#### Control

During the control trial participants sat and rested for 30 min.

### Day 2

Subjects arrived at 8:45 am and rested for 15 min before a cannula (20G) was inserted into a vein in the antecubital fossa, and a baseline blood sample was collected. The cannula was flushed regularly with saline throughout the day. A standardized high-fat meal was then consumed for breakfast. This consisted of white bread, mayonnaise, butter, whole milk, cheddar cheese and potato crisps. This meal provided 812±96 kcal energy with 56% from fat, 33% from carbohydrate and 11% from protein. The meal contained 0.7 g fat, 1 g carbohydrate, 0.3 g protein and 11 kcal per kg bodyweight. The mean macronutrient content of the meal was 51.2±10.8 g fat, 68.4±14.4 g CHO and 23.3±4.9 g protein. The mean time taken to consume the meal was 10 min 58 s±3 min 24 s. 

Further blood samples were collected 1, 2 and 3 hours after the breakfast meal. A second identical meal was then consumed for lunch with subsequent blood samples taken 4, 5, 6, and 7 hours after the consumption of the first meal. Water was provided *ad libitum* throughout the day of the first trial and this volume of water was consumed during the subsequent trial. Upon completion of day 2 participants were asked to rest for the remainder of the day, consume an evening meal (which was recorded and replicated during the subsequent trial) and fast from 10 pm (water allowed) before returning to the laboratory for day 3. 

### Day 3

Subjects arrived at 8:45 am and the protocol for day 2 was repeated as day 3. 

### Measurements

#### Blood handling and analysis

Blood samples were collected with sterile 6 ml K+EDTA non-ridged vacutainers (Vacuette, greiner bio-one, Kremsmunster, Austria) and were centrifuged (Eppendorf Centrifuge 5702/R, UK.) at 3500 rpm at 4 °C for 10 min. Then plasma was removed and frozen at -20 °C until analysis. Plasma TG (CV = 0.81%) and glucose (CV = 2.22%) concentrations were assessed using manual enzymatic colorimetric assay kits (Randox,Crumlin, Co. Antrim, UK) using a spectrophotometer (Camspec M330B, Leeds, UK). 

#### Ex vivo Immune Activation

Sample preparation and analysis was carried out on the day of sample collection with the cytometer settings the same for every analysis performed. The expression of CD11b on leukocytes was determined by Alexa Fluor-488 mouse anti-human CD11b and APC mouse anti-human CD36 used to determine CD36 expression. As negative controls Alexa Fluor-488 mouse IgG1, k isotype and APC mouse IgM, k isotype controls were used. Measurements of leukocyte activation were performed on fasting, 2, 5 and 7 hours blood samples from days 2 and 3 of each trial. 5 µL of Alexa Fluor-488 and 20 µL of APC was added to a FACS tube prior to adding 100 µL of whole blood. Samples were then incubated for 30 min in a darkroom at room temperature. Red blood cells were then lysed by adding 2 ml of pharmlyse solution and further incubated for 15 min. Samples were centrifuged for 5 min (250 G, 4 °C), washed in PBS (0.2% BSA) and centrifuged again before being fixed in 0.5 ml of 1% paraformaldehyde in PBS. For the negative controls 100 µL of whole blood was added along with each isotype control (CD11b and CD36), and 100 µL of whole blood was used as an unstained control. 

Samples were analysed using a FACS calibur (BD, Oxford, UK) and the data processed using FlowJo software. 50,000 cells were analysed for each sample. Granulocytes, monocytes, and lymphocytes were identified using forward and side scatter plots and mean fluorescent intensity (MFI) measured to indicate the surface expression quantity per leukocyte. The between day coefficient of variation for CD11b was 8.5% and for CD36 it was 7.6%. 

#### Determination of LTTH

A sample group of 5 subjects was used for this assay, where we had sufficient volume of frozen plasma to carry out this analysis. Analysis was carried out in a batch at the end of the study. Spontaneous lipolytic activity in TRLs resulting from LPL bound on their surface was measured using the LTTH assay as previously described [26]. TRLs were isolated by fast-protein liquid chromatography (FPLC) using a Superose 6 HR 10/30 column (Pharmacia) at 4 °C to separate lipoproteins in TSE buffer containing 10IU/ml heparin for stabilisation of LPL during the procedure. 1 ml of filtered plasma was applied to the column and chromatographed at a flow rate of 0.3 ml/min under a pressure of 150 psi. Fractions corresponding to total TRLs, including both chylomicrons and VLDL (fractions 9 to 18), were pooled and immediately assayed for LPL activity. Aliquots of the pooled TRLs corresponding to 0.3 μmol of TG were incubated for one hour at 37 °C of buffer and lipolysis monitored over time. Blanks were obtained by incubations of samples in the presence of 2 mmol/L Paraoxon (Sigma), which totally blocks LPL activity. The resulting amounts of non-esterified fatty acids (NEFA) released by LPL bound to TG rich lipoprotein were then measured in triplicate, and after correction for plasma TG concentrations, LTTH was finally expressed as the amount of NEFA released per ml of plasma per hour. The coefficient of variation, for duplicate samples, for the LTTH assay was 7.3%.

### Statistical analysis

Data were analysed using the GraphPad Prism 5 software. Normality was checked using the Shapiro-Wilk test, with no data requiring transformation. Both total and incremental (taking into account changes in baseline concentrations) area under the curve (AUC) values for plasma TG and glucose concentrations were calculated using the trapezium rule. The AUC values were calculated to provide a summary of the TG and glucoses responses during the 7 h test period on each day. Calculated incremental and total AUC values on each day were compared between trials using a two-way ANOVA. To compare differences between the trials over time a two-way ANOVA with repeated measures was performed. Where a significant effect was observed Bonferroni's Multiple Comparison Tests were performed to locate differences. Significance was taken at P<0.05. Results are presented as means±S.E.M. 

## Results

### Power Output and Energy Expenditure

The average peak power output during HIIE was 870.3 ± 140.4 W and the average mean power output was 464.3 ± 119.7 W. This corresponded to an estimated energy expenditure of 93.1 ± 20.1 kcal during HIIE.

### Plasma Glucose and Triacylglycerol

**Figure 1:**
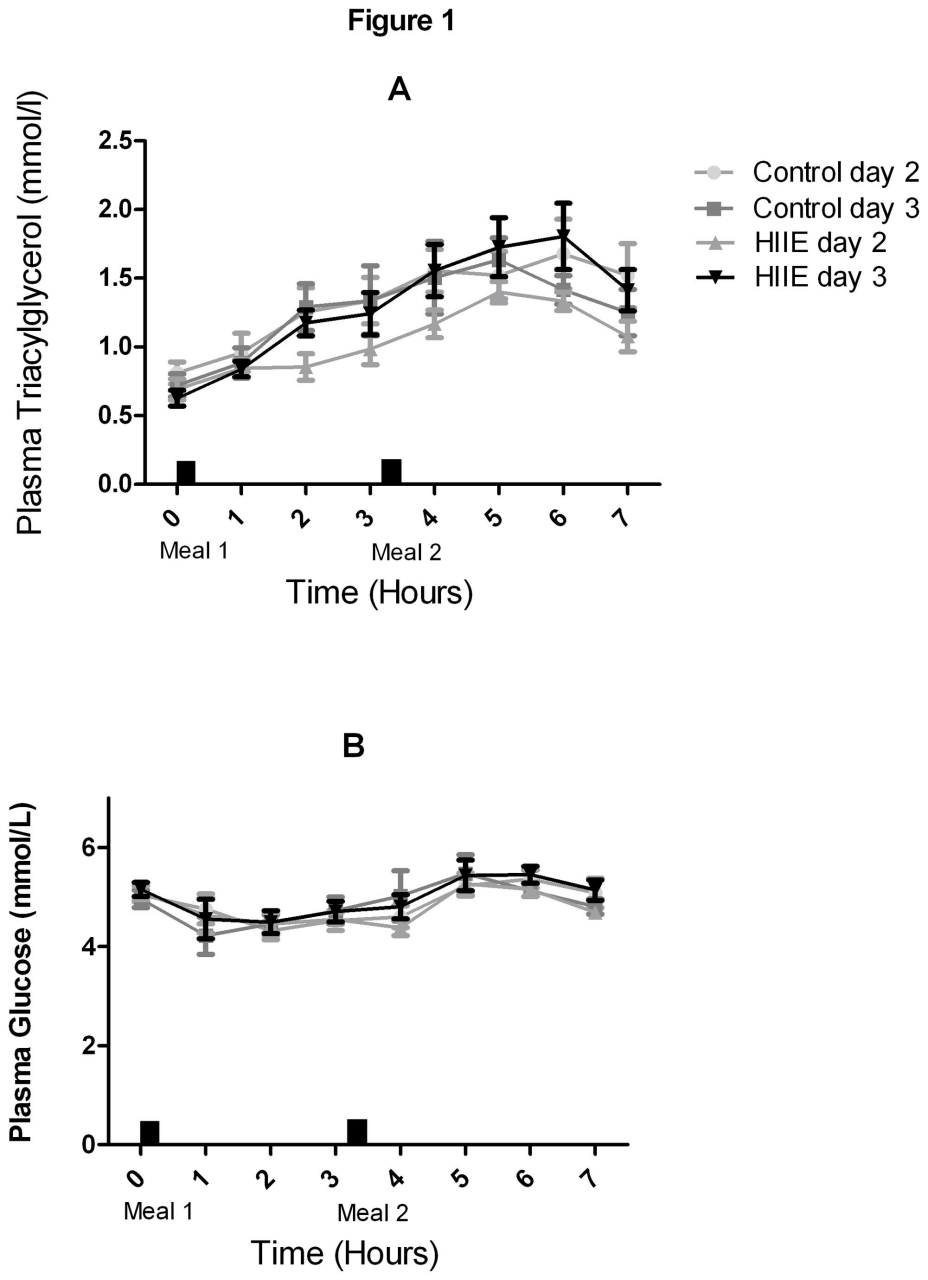
Plasma TG(A) and glucose (B) concentrations on day 2 and 3 of the HIIE and control trials.

Plasma TG and glucose values over time during control and HIIE trials are presented in Figure 1. There were no differences between the trials in plasma glucose total and incremental AUC. When comparing TG total AUC this was lower (P<0.05) on day 2 of HIIE compared to day 2 of the control trial (Table 1). There were no differences in total AUC between HIIE and control groups during day 3 of the trial. Analysis of variation revealed a significant (P<0.05) group effect for incremental TG AUC, however Bonferroni post hoc tests found no differences between HIIE or control trials on either day 2 or day 3. 

**Table 1:** TG total and incremental AUC over the 7 h experimental period of day 2 and 3 of the HIIE and control trials.

	**Control**		**HIIE**	
	**Day 2**	**Day 3**	**Day 2**	**Day 3**
**Incremental TG AUC (mmol/l/7h)**	3.87±0.68	4.01±0.67	2.64±0.15	4.98±0.75
**Total TG AUC (mmol/l/7h)**	9.47 ±1.07	9.36±1.07	7.46±0.54*	9.05±0.92

^*^ denotes a significant difference (P <0.05) from day 2 of the control trial.

### Ex. *Vivo* Immune Activation

**Figure 2:**
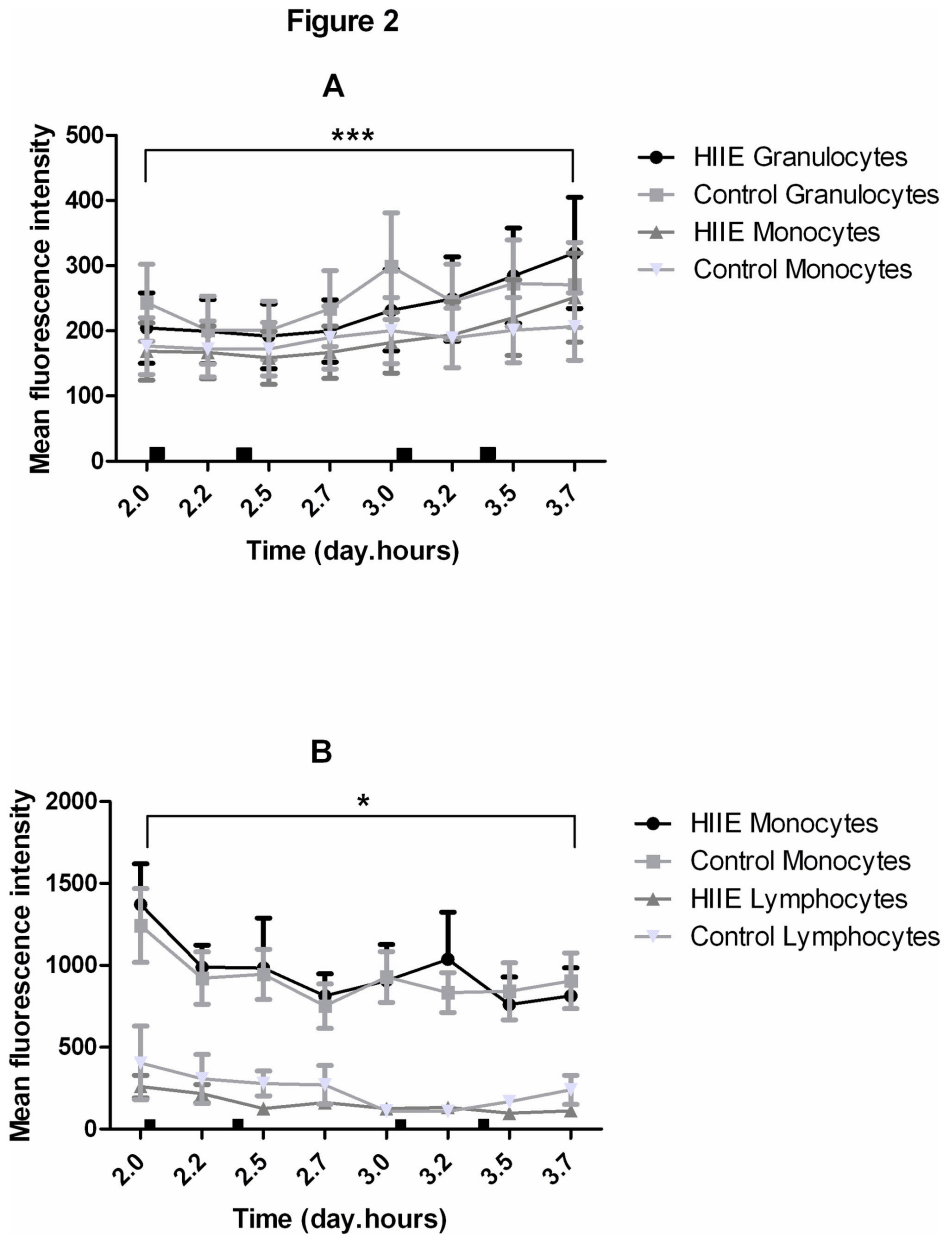
CD11b (A) and CD36 (B) Mean fluorescence intensity on gated cell populations on day 2 and 3 of the control trials. (A) *** denotes an effect of time for granulocytes and monocytes (P<0.001) (B) * denotes an effect of time for monocytes (P<0.005) and lymphocytes (P<0.05). ■ denotes laboratory meal times.

Analysis of variance revealed CD11b expression increased with time over day 2 and day 3 of the study in lymphocyte (P<0.0001), monocyte (P<0.0001) and granulocyte (P<0.0001) populations (Figure 2A). Lymphocyte and monocyte CD36 expression decreased with time over day 2 and day 3 of the study (P<0.05) (Figure 2B). There were no group or interaction effects noted in either CD11b or CD36 expression amongst all leukocyte subgroups. 

### LTTH Assay

**Figure 3:**
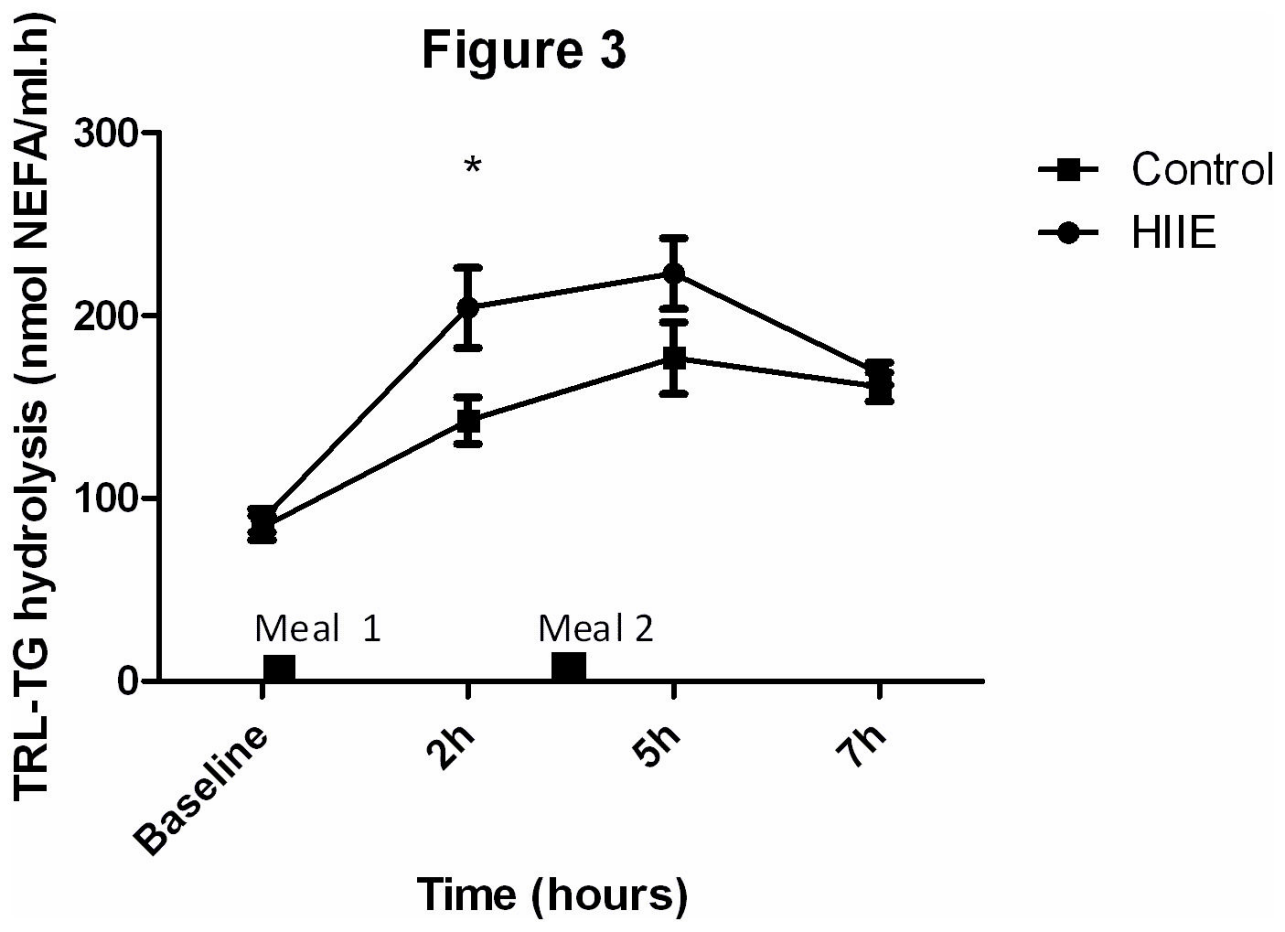
LTTH activity on day 2 of the HIIE and control trials. * denotes a significant (P<0.05) difference between HIIE and control trials.(N=5).

Analysis of variance revealed an interaction and time (P<0.05) effect in LTTH activity, with no group effect (P=0.09) observed. Post hoc tests showed that LTTH activity was higher (P<0.05) at 2 hours on day 2 of HIIE (204.3±31 nmolNEFA/ml.h) compared to control trial (142.7±17.9 nmolNEFA/ml.h) (Figure 3).

## Discussion

The main findings of the present study were that HIIE attenuated postprandial TG on day 2 of the study compared with a control trial, with this effect abolished by day 3. The attenuation of postprandial TG the day following HIIE is supported by recent studies [22,23] and the current study extends this finding by demonstrating that this reduction in plasma TG is associated with an increase in LTTH. Prior to the current study the longevity of, or mechanisms underlying, these effects had not been investigated. 

The novel finding of the current study, that the beneficial effect of HIIE on postprandial TG is abolished by day 3, suggests that one must engage in HIIE every 1-2 days to attenuate the rise in postprandial TG which is a chronic risk factor for CHD [4]. Previous studies have proposed that the effect of endurance exercise on postprandial TG is acute, rather than a training effect [27][28] [29], with the current study indicating the same is true for HIIE. On top of this benefit of HIIE, in reducing postprandial TG, training studies ranging from two to six weeks in duration have shown that HIIE can improve endurance performance, muscle oxidative capacity [30], insulin action [31] and endothelial function [32]. It is also possible that more chronic improvements in insulin sensitivity, observed after HIIE, would also result in long term reductions in TG levels, however, this remains to be investigated. Whilst similar benefits have been found after more prolonged endurance training HIIE may also be beneficial as a public health tool. As time is often cited as the primary barrier to exercise participation [24] the use of such a protocol has therefore been proposed to overcome this barrier. Whilst the total exercise time in the current study was only 2.5 min the session did last ~25 min, which is slightly shorter than the current recommendations, it remains to be investigated whether this would increase exercise participation. In that regard, although using a different protocol (6*3 min at 90% VO_2max_), high intensity interval training been found to be more enjoyable than standard endurance training [33] suggesting adherence may be improved, although this needs to be examined using the HIIE protocol employed in the current study.

The mechanisms responsible for the reduction in postprandial TG concentrations after exercise remain to be elucidated. Previous studies using moderate intensity, continuous exercise have shown an upregulation in LPL activity for up to 18 hours, an observation associated with a fall in serum TG after exercise bouts lasting several hours [34][35]. Some studies have suggested that there is a threshold energy expenditure above which LPL activity is increased following moderate intensity exercise. A study comparing 4 separate bouts of moderate intensity exercise with energy expenditures of 800, 1,100, 1,300 or 1,500 kcal, found that the 800 kcal exercise induced a reduction of postprandial TG, but there was no increase in LPL activity [36]. On the other hand the 1,100, 1,300 and 1,500 kcal exercise bouts showed a significant increase in LPL activity 24 hours after the bout of exercise, again with a reduction of postprandial TG. Further studies have shown that moderate intensity, continuous exercise (215-1075 kcal energy expenditure) reduces postprandial TG but does not induce a statistically significant increase in LPL activity [37] [38] [39]. In light of these studies showing a reduction of postprandial TG with no increase in LPL activity, it has been proposed that a decreased hepatic VLDL secretion also plays a role in reducing postprandial TG after moderate intensity exercise, particularly where energy expenditure is low (for review see 40). Previous studies have shown indirect evidence of this effect on hepatic vLDL secretion after moderate intensity exercise [41]. Further support of this comes from the same lab where moderate intensity exercise had no effect on TG clearance, monitored after intralipid infusion [42]. On the other hand further study has shown that there was no effect of moderate intensity exercise on hepatic VLDL-TG secretion rate [43][44]. It seems then, that moderate intensity exercise may attenuate postprandial TG via an increased LPL activity (possibly above a threshold energy expenditure), with conflicting evidence as to whether decreased hepatic VLDL-TG secretions play a major role. However, the mechanisms of reducing postprandial TG have not been studied after HIIE in humans. It is worth noting, at this point, that the current study measures LTTH in pre-heparin LPL which is more likely to reflect physiological processes rather than studies employing heparin infusion where the majority of the LPL is released. 

There is some evidence in rats that muscle contractile activity is the main regulator of LPL activity [45]. This study also indicated that the intensity of physical activity required to facilitate an upregulation of LPL activity may be fibre type specific with LPL activity, following high intensity exercise, increasing only in fast fibres. Additionally, increases in LPL activity seemed to be due to local contractile activity rather than hormonal changes [46], although these findings cannot be directly applied to humans. In our previous study, using HIIE [23], we found that β-hydroxybutyrate was not changed after high intensity exercise. This would suggest that a change in VLDL secretion rate was not responsible for the reduction in postprandial TG observed after HIIE, although LPL activity was not measured in the previous study. In the current study, lipolysis in TRLs resulting from LPL bound on their surface was measured using the LTTH assay as previously described [26]. LTTH was greater after HIIE at 2 hours of day 2 of the trial, compared with the control group, indicating that the main mechanism through which HIIE reduces postprandial TG is an increase in TRL bound TG hydrolysis by LPL and subsequent peripheral tissue, most likely skeletal muscle, uptake. This increase in LPL dependent TG hydrolysis could be due to an increase in LPL activity and/or an increased affinity of TRL for LPL. Indeed recent work has indicated that after moderate intensity exercise there may be compositional changes to VLDL_1_ particles, by increasing particle size, that may increase affinity to LPL for clearance from the circulation [47]. Whether such a change occurs after HIIE merits further examination. 

If it is the case that LPL activity is increased by HIIE then this is would be somewhat surprising if HIIE is viewed in light of the hypothesised energy expenditure threshold proposed for moderate intensity exercise. The estimated energy expenditure of HIIE in the current study is only 93.1 ± 20.1 kcal. Even allowing for a large error in estimation or post-exercise oxygen consumption this is well below the proposed threshold of 1,100 kcal for moderate intensity exercise. It is, therefore, possible that LPL activation is fibre type specific in humans, as well as rats, and that if exercise is of sufficient intensity/duration to recruit fast fibres then LPL activity will be increased. Further work is clearly required to firstly confirm the hypothesised increase in LPL activity after HIIE and secondly to investigate the potential for this to be a fibre type specific effect. 

As many of the deleterious effects of elevated postprandial TG are thought to be due to leukocyte activation we investigated whether HIIE could attenuate this. We found that there was no difference in any of the markers of leukocyte activation used in the current study between trials, although effects of time were noted. The rise in CD11b during day 2 and 3 of the study in monocyte, lymphocyte and granulocyte populations is likely due to the rise in postprandial TG, as increased TG levels after a high-fat meal have previously been shown to activate leukocytes and increase CD11b expression [13]. This may be caused by endothelial inflammation which is enhanced in response to lipid exposure. The majority of this increase was seen in day 3 and it may be that this more prolonged, compared to a single high fat meal (ie early on day 2), level of exposure to lipids is required for this immune activation to occur. Further work designed to specifically investigate this is needed. To the best of our knowledge, the current study is the first to assess CD36 expression in human leukocytes after a high-fat meal. The current study showed that CD36 expression was decreased in lymphocyte and monocyte populations over day 2 and 3 of the study. This decrease in CD36 surface expression might indicate increased CD36 internalization as Zamora et al 2012 [10] have shown that CD36 can become internalized in response to a pro-inflammatory environment. Furthermore, it is believed that decreased expression of CD36 in macrophages, induced by various cytokines, is linked to the development of atherosclerosis [48][49], possibly due to Toll-like receptor (TLR) pathway activation arising from the increase in cytokines, such as TNF-α. This can occur during atherogenic conditions, often seen after a high fat meal [10], where CD36 takes up and internalises oxLDL and may eventually lead to foam cell formation in macrophages [48]. While the current study found no effect of HIIE on markers of leukocyte activation only a limited number of markers, from a wide number available, were monitored and clearly further work is needed in this area. Indeed a recent study found that moderate intensity exercise blunted the postprandial rise in the markers of leukocyte activation CD11a and CD18 [50], however this has not been investigated after HIIE. 

In conclusion, the beneficial effects of HIIE on postprandial TG are abolished 2 days after exercise. This suggests that although high intensity exercise may be a time-efficient tool that is useful in the prevention of CVD, it will have to be performed 3-4 times a week to be effective. Furthermore, whilst the current study demonstrates a reduction in postprandial TG after HIIE, this is in young healthy participants. Whilst this exercise may be of benefit in this group in the prevention of future disease there is a need to carry out such studies in populations who are at risk of CVD, such as in obesity or type 2 diabetes. This reduction in postprandial TG after HIIE was associated with an increase in LTTH activity, with no difference in markers of leukocyte activation between trials.
